# Virtual instrumentation for electro–analytical measurements

**DOI:** 10.1155/S1463924699000061

**Published:** 1999

**Authors:** A. S. Economou, G. J. Volikakis, C. E. Efstathiou

**Affiliations:** Laboratory of Analytical Chemistry Department of Chemistry University of Athens Panepistimiopolis Athens 157 71 Greece

## Abstract

This paper deals with some applications of Virtual Instrumentation to electroanalytical measurements. Virtual Instruments (VIs) are software programmes that simulate the external appearance and functions of a real instrument on the screen of a computer. In this work, programmes have been developed to control the potential of a working electrode (through a suitable potentiostat), acquire the current response, process the acquired current signal, and control a peristaltic pump and injection valve. The sequence of operations was controlled by the VI. The programmes developed have been applied to amperometric and voltammetric measurements in static and flowing solutions. The Vl package that has been used was Lab VIEW 4.0.1 from National Instruments.


Journal of Automated Methods & Management in Chemistry, Vol. 21, No. 2 (March–April 1999) pp. 33–38

### Virtual instrumentation for electro-analytical measurements

**A. S. Economou, G. J. Volikakis and C. E. Efstathiou**

*Laboratory of Analytical Chemistry, Department of Chemistry, University of Athens, Panepistimiopolis, Athens 157 71, Greece*

*This paper deals with some applications of Virtual Instrumentation to electroanalytical measurements. Virtual Instruments (VIs) are software programmes that simulate the external appearance and functions of a real instrument on the screen of a computer. In this work, programmes have been developed to control the potential of a working electrode (through a suitable potentiostat), acquire the current response, process the acquired current signal, and control a peristaltic pump and injection valve. The sequence of operations was controlled by the VI. The programmes developed have been applied to amperometric and voltammetric measurements in static and flowing solutions. The VI package that has been used was LabVIEW 4.0.1 from National Instruments.*

### Introduction

Virtual Instrumentation is a concept that encompasses any software system that tries to simulate the external appearance and internal functionality of a real-world instrument on the screen of a computer. Any program that fulfils this requirement is called a Virtual Instrument (VI) [1, 2]. In most cases of commercial systems, the VI concept is based on an object-oriented programming language. The introduction and development of such VI systems has been prompted by the requirements of modern scientific instrumentation. More specifically, the advantages of VI systems are the added versatility that comes with software, the reduced cost compared to dedicated real instruments, the ease of customization to each user's specific needs, and the intrinsic user friendliness of VIs.

When applied to analytical problems, VI systems, in common with other instrumentation systems, are expected to perform the following tasks: (i) provide an excitation signal that perturbs the system under study; (ii) measure the response of the system to the perturbation; (iii) process and display the acquired signal; and (iv) perform auxiliary functions related to the process of measurement.

In this work, the scope of different VI systems for electroanalytical chemistry was exploited. In particular, the electroanalytical techniques that have been assessed were amperometry with Flow Injection Analysis (FIA), cyclic voltammetry (CV) and on-line adsorptive stripping voltammetry (AdSV). Amperometry is the simplest electroanalytical technique involving the application of a constant potential on a working electrode. Provided that the value of the potential is high enough to initiate a redox reaction, the analytical species is reduced or oxidized. This electrochemical process results in a current flowing through the cell. The magnitude of the current is related to the analyte concentration in the sample. When combined with FIA, in which discrete injections of a sample are made, characteristic peaks are obtained in the current–time response. Cyclic voltammetry is based on the application of a triangular potential–time waveform on the working electrode while the current flowing through the cell is recorded. AdSV is a complex electroanalytical technique relying on a preconcentration stage by adsorption of the analyte on the working electrode. Its on-line version makes use of a flow system and flow-through cell to deliver the sample to the working electrode. In this work, complete VIs have been developed to control the various stages of the measurement as well as all of the equipment required for the experiments.

### Experimental

### *Software*

The software platform that was used throughout this work was LabVIEW Version 4.0.1 (National Instruments, TX) [3–5]. This is a programming tool combining VI with object orientation. In LabVIEW, a VI is composed of two parts: (i) the front panel, which is the user interface of the programme, and simulates the appearance and functionality of a real instrument. The front panel is composed of controls (push buttons, sliders, control knobs, etc.) through which the user inputs data and commands to the programme and indicators (chart recorders, graphs, gauges, etc.) that display the results of an experiment. LabVIEW, also, provides libraries of data manipulation icons (e.g. statistics and filters). The front panel is built by inserting the appropriate controls and indicators in a drag-and-drop manner on the front panel frame; (ii) the block diagram, i.e. the part of the VI in which the actual programming is carried out. As soon as a control or indicator is placed on the front panel, a 'shadow' block representing it is automatically inserted in the block diagram. The control and indicator blocks are combined with different operators (adders, differentiators, etc.) which are also drag-and-dropped as blocks and 'wired' together to perform different operations. Each VI can be incorporated into a larger VI as subVI in a similar way to a subroutine. Therefore, the complexity of the overall VI could be increased maintaining an instrumental hierarchy.

Graphical programming in LabVIEW replaces conventional text-based code with icons wired together. Instead of laboriously typing blocks of text, with the associated risk of syntactical and logical errors, LabVIEW relies on connecting a few icons with wires. In addition, most users, including experienced ones, find it intuitively easier to visualize and interpret icon-based rather than text-

1463-9246/99 $12·00 © 1999 Taylor & Francis Ltd based programmes. This results in a user-friendly environment with shorter familiarization and development times, and leads to easier debugging and modification. On the other hand, speed, data manipulation potential, presentation capabilities and interfacing flexibility are, at least, the equal of any conventional programming language. Most users find that it only takes a fraction of the time and effort to develop a programme in LabVIEW than in conventional languages, not to mention future modifications and alterations in which LabVIEW excels.

### *Hardware—Apparatus*

For the electrochemical experiments, two potentiostats were used: (i) a home-made adder-type potentiostat [6], mainly used for amperometry and DC voltammetry; and (ii) a Tacussel PRG5 polarograph used for DP voltammetry. For the FIA and AdSV experiments, a Rheodyne type 50 six-way rotary injection valve controlled by a Rheodyne 5701 pneumatic actuator and a Gilson Minipuls3 peristaltic pump were employed. The valve was set up in a two-way configuration allowing either the sample or carrier solution to be drawn by the pump. A home-made auxiliary circuit was built to drive the pneumatic actuator of the injection valve under TTL control. Also for FIA, a commercial Metrohm 641-VA wall-jet electrochemical flow cell equipped with an Au counter electrode and an Ag/AgCl reference electrode was used. For batch measurements, a home-made cell with a Pt counter electrode, Ag/AgCl reference electrode and magnetic stirrer were employed.

The instruments were interfaced to a National Instruments LABPC+ card. This is a data acquisition and I/O card featuring: four differential (or eight single-ended) 12-bit ADCs with a maximum sampling rate of 83 kHz, software selectable gain (1–100), and unipolar or bipolar operation (0–10 V or −5 to +5 V, respectively); two 12-bit DACs with unipolar or bipolar operation (0–10 V or −5 to +5 V, respectively); 24 digital I/O lines with TTL compatibility; and three 16-bit counters with a 2 MHz base clock rate. The card supports direct memory access (DMA) transfers. It was installed in a Zenith Z-Select 100 486 PC with LabVIEW 4.0.1 running under Windows 3.1. In this work, all the ADCs were connected in the differential configuration.

All the electrodes were made of carbon paste (called carbon paste electrodes or CPEs), prepared by thorough mixing of dry graphite powder (Fluka) and a pasting liquid. Two types of pasting compounds were used: (i) nujol (Merck, IR grade, 5 + 3 m/m) for the NU-CPE electrode; and (ii) diphenylether (Fluka, 5 + 2 m/m) doped with tri-*p*-cresylphosphate (Merck, 1% w/v in the ether) to keep the pasting agent liquid, for the TCP-CPE electrode. The CPEs were fitted in the flow-through cell facing the inlet in a wall-jet configuration.

Two experimental configurations were developed for this work. The first one was used for cyclic voltammetry and made use of the home-made potentiostat and the batch electrochemical cell. The set-up is shown in figure 1(a). The second configuration was used for amperometric detection in FIA and made use of the home-made potentiostat, and for on-line AdSV making use of the commercial potentiostat. This set-up is illustrated in figure 1(b).

### *Procedure*

*Cyclic voltammetry of potassium cyanoferrate(III).* A solution of potassium cyanoferrate(III) in 1.0 M KCl as supporting electrolyte was chosen for the cyclic voltammetry experiment due to its well-known redox reversibility in aqueous solutions.

*Flow injection analysis of potassium cyanoferrate(III).* A solution of potassium cyanoferrate(III) was prepared in 1.0 M KCl as supporting electrolyte and transferred into the sample reservoir. Injections of this solution were made into the flowing carrier and the current was recorded at an operating potential of +0.20 V versus Ag/AgCl. The injection volume was 0.3 ml.

*Determination of rutin by differential pulse voltammetry.* The rutin solution in Britton–Robinson (B–R) buffer pH 5.0 was injected in the carrier B–R buffer solution, adsorbed on the CPE surface at +0.20 V and, finally, quantified by cathodic differential pulse voltammetry in stationary solution (pulse amplitude 50 mV and pulse period 2 s).

### Results and discussion

### *Cyclic voltammetry*

For this experiment, the home-made potentiostat was used. The triangular potential waveform was created in software and imposed on the working electrode through one DAC output. The voltage output of the DAC was in the range ±5 V, and a voltage divider at the input of the potentiostat converted the potential range to a more useful ±1.8 V. Accordingly, the potential resolution was altered from 2.4 mV to 0.8 mV. However, 1 mV potential increments were used to generate the potential ramp. Since 1 mV steps were used, the DAC scan rate (in samples/s) numerically coincided with the selected potential scan rate (in mV/s).

An ADC input was used to acquire the current data. The ADC sampling scan rate was the same as the DAC scan rate (i.e. one current value was sampled for each 1 mV potential increment). It must be noticed that, in fact, due to the digital character of the potential waveform, the technique is staircase cyclic voltammetry, which at the limit of very small potential increments approaches DC cyclic voltammetry. Real time display of the response was only possible for scan rates up to 50 mV/s owing to limitations in updating the display graph. For higher scan rates, the data were saved in a buffer and displayed post-experimentally. The front panel of the programme for cyclic voltammetry, CV.VI, is illustrated in figure 2, which shows a voltammogram for cyanoferrate (III).

The data could be loaded in a processing VI, LOADCV.VI, that performed the following functions: (i) smooth the signal using a curve-fitting procedure; (ii) calculate the peak positions in both forward and reverse scans; (iii) measure the peak heights after selecting a

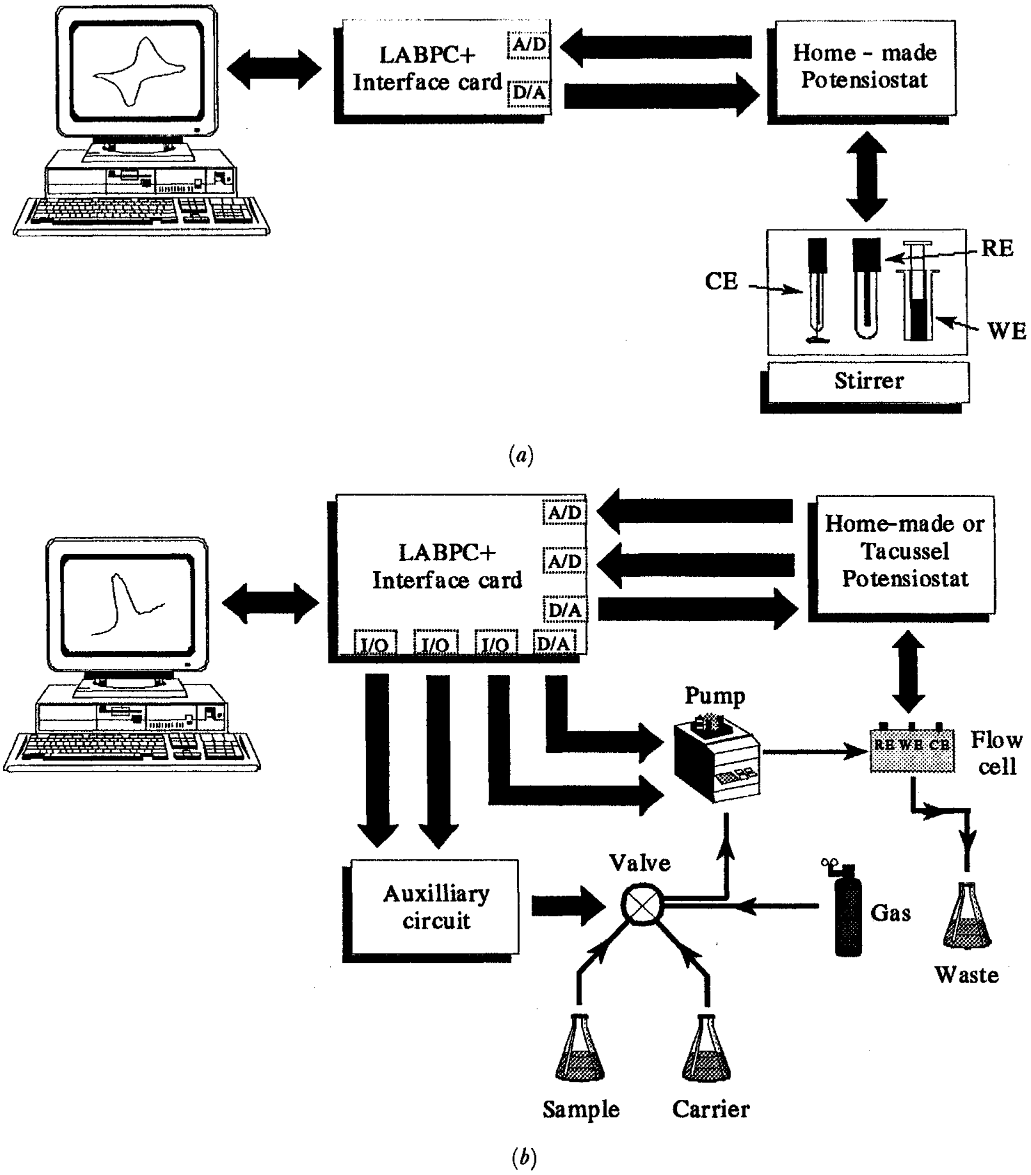

*Figure 1. The set-up and operating principle of: (a) cyclic voltammetry configuration; (b) amperometry with FIA and AdSV configuration.*

baseline by using the cursor; and (iv) display the results of the calculations.

### *Amperometry with FIA*

The constant potential was imposed on the working electrode through one DAC output. The current was sampled through an ADC input. The DAC sampling rate was usually 2 samples/s. The pump was turned on and off by means of a TTL signal through an I/O line while its speed was controlled through the second DAC output. Two additional I/O lines were used for turning the valve on and off. The front panel that controlled this experiment is shown in figure 3. More specifically, the AMPER.VI set the potential applied to the working electrode through a turning knob. The potential could be set in the range $\pm 1.8$ V with a resolution of 0.8 mV. The VALVE.VI controlled the timing of the injection valve, i.e. the number of injections required, injection time and time between injections (interval time). The GLOB1.VI contained status LEDs to display the current position of the valve. The PUMP.VI controlled the operation and speed of the pump. The ACQUIRE.VI recorded the current response. Limits were set that define the width of the peak so that integration of the peak could be carried out. In figure 3, acquired data are displayed for a sample containing cyanoferrate(III).

Once the response was recorded, the LOADFIA.VI, existing as a subVI in the ACQUIRE.VI, accepted the data and processed them. The number, position and height of the peaks were displayed on the screen. Also, each individual peak could be displayed on the screen with information on its properties.

### *On-line adsorptive stripping voltammetry*

A DAC output connected at the appropriate terminal of the polarograph was used as an auxiliary external potential to the working electrode; this was used to bypass

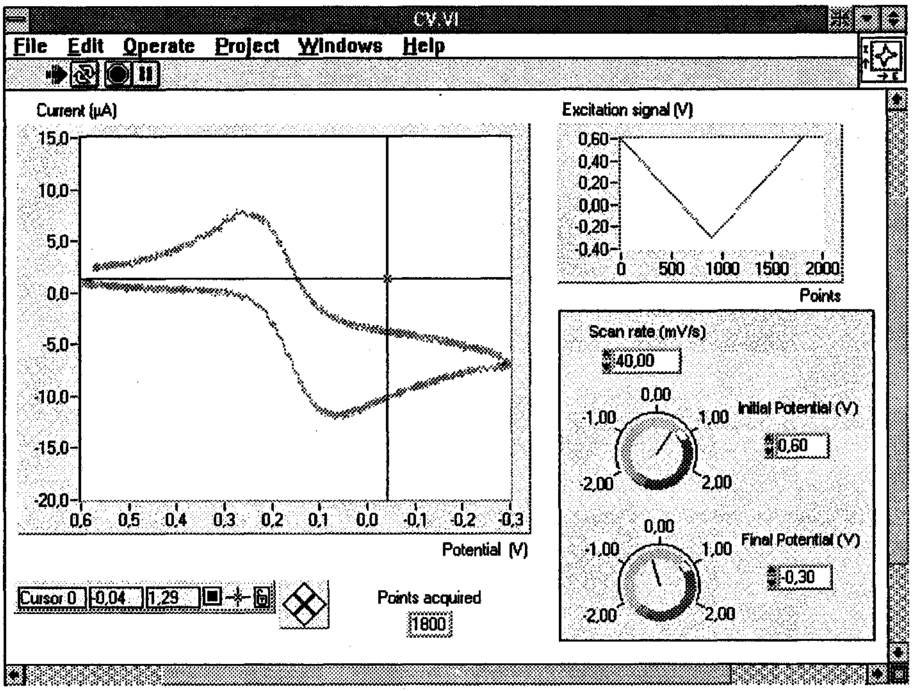

*Figure 2. LabVIEW front panel for cyclic voltammetry. This VI is shown in the process of obtaining the voltammogram of an aqueous potassium cyanoferrate(III) (1.00 mmol $l^{-1}$) solution.*

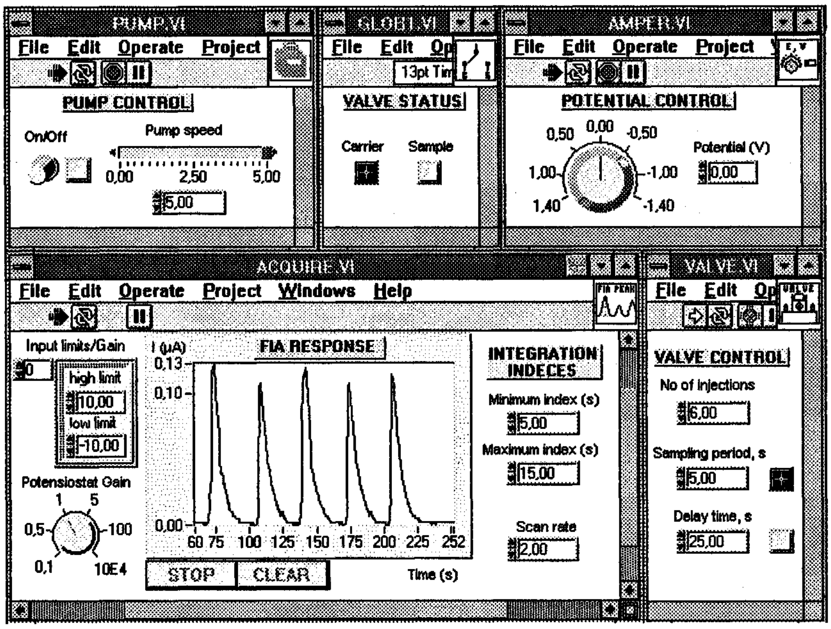

*Figure 3. LabVIEW front panel for the amperometric detection in FIA. This VI is shown in the process of obtaining FIA peaks of an aqueous potassium cyanoferrate(III) (1.00 mmol $l^{-1}$) solution.*

the limitation of the fixed potential scan ranges of the Tacussel polarograph. Both the potential and current recording were performed by the analogue potentiostat circuitry, but their values were sampled through two ADC inputs connected to the appropriate polarograph terminals for display of the current–potential plot. Sampling and recording of the current–potential response started and ended as soon as the potential had reached the initial and final potential values. The data were displayed in real time at a rate of 1 sample/s. The front panel that controlled the experiment is shown in figure 4.

The main programme was the STRIPDP.VI that contained controls relevant to the measurement, and the two other VIs, DP.VI and STRIPGLOB.VI, were sub-VIs, as named by the main programme. The preconcentration time and potential, pump speed, cleaning time and potential, pretreatment time and potential, and medium exchange time were user defined. The STRIPDP.VI controlled the pump through a DAC (pump speed) and TTL line (for starting and stopping the pump). The valve was controlled through two TTL lines (to switch between sample and carrier). The control 'starting po-

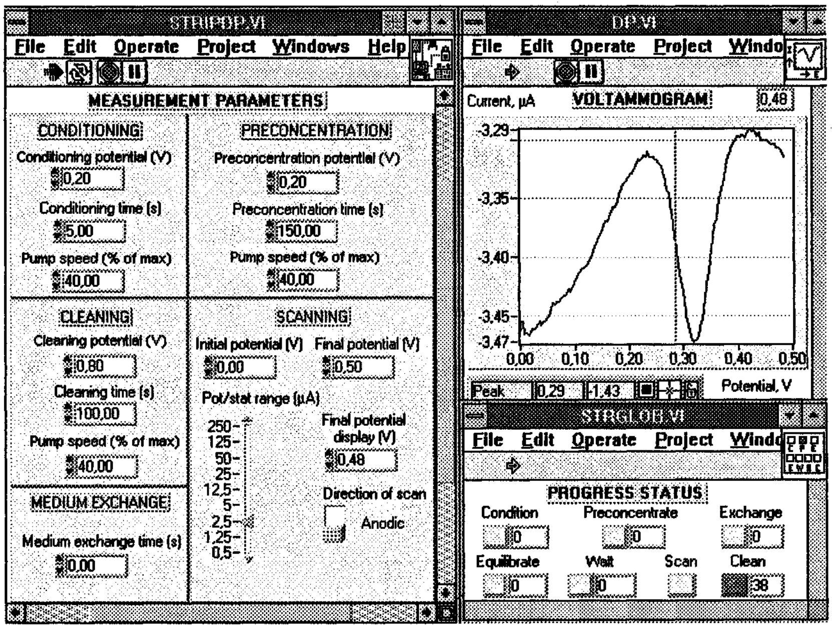

*Figure 4. LabVIEW front panel for adsorptive stripping voltammetry. This VI is shown in the process of obtaining the voltammogram of an aqueous rutin (1.00 × $10^{-8}$ mol $l^{-1}$) solution.*

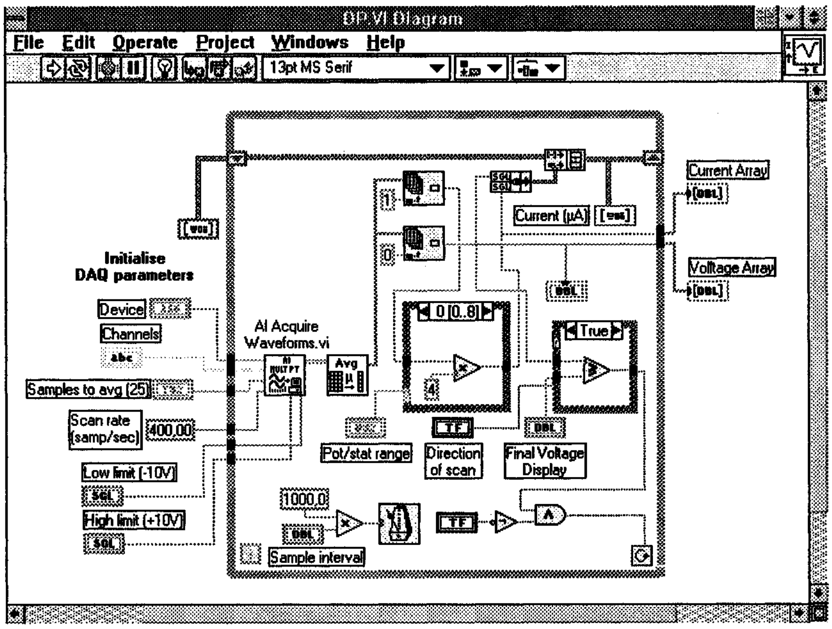

*Figure 5. Block diagram of the DP.VI, a sub-VI that acquires and displays the current–potential response.*

tential' was used to synchronize the start of the sampling with the start of the scan, while the 'range' is the range set on the potentiostat and scales the current. The DP.VI acquired potential and current data, and plotted them on a graph. The block diagram of DP.VI is illustrated in figure 5. In this diagram, all the icons were inside a 'while' loop, which was repeated at an interval defined by the control 'sample interval' (in this case 1000 ms or 1 s). The 'AI Acquire Waveforms.VI' was a sub-VI that acquired the potential and current data (from two separate ADC channels 1 and 2, respectively). Since the 'AI Acquire Waveforms.VI' was repeated every second, the actual effective sampling rate was 1 sample/s. In order to reduce noise, signal averaging was employed. Twenty-five samples (as indicated by the 'samples to average' control) were acquired from each channel at a faster 'scan rate' of 400 samples/s. The 'AI Acquire Waveforms.VI' was wired to the 'Avg' block which is a sub-VI that performed the actual averaging of the 25 sample data acquired. The output of the 'Avg' block (which was an array) was fed into two array manipulation blocks that separated the current and voltage values. The current magnitude was multiplied by a value controlled by the 'potentiostat range' control in order to scale the current in microamperes. Then, the current and voltage values were input to the 'current (1 µA)' indicator, which was a graph that displayed the current–potential response and which was updated every second.

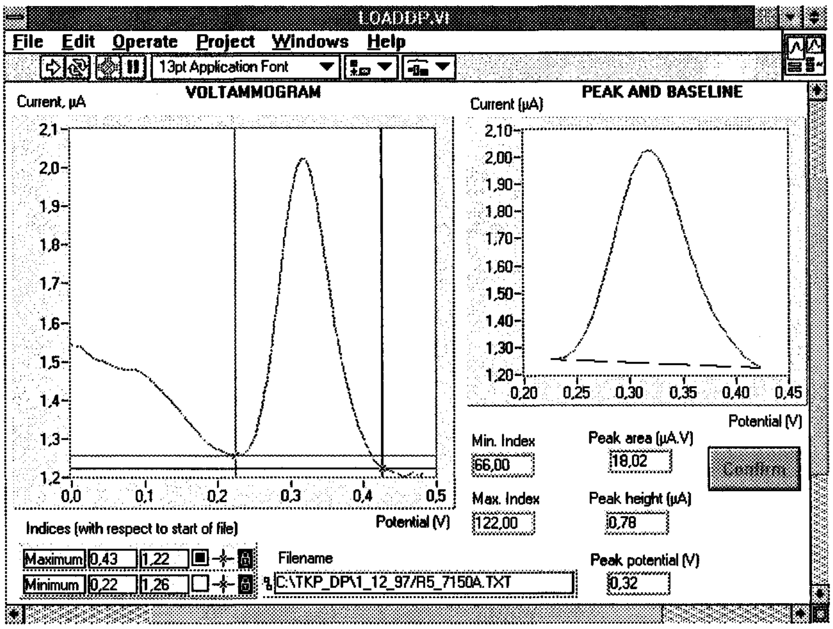

*Figure 6. LabVIEW front panel for processing voltammograms obtained by AdSV. This VI is shown while processing the voltammogram of an aqueous rutin (5.00 × $10^{-8}$ mol $l^{-1}$) solution.*

The 'while' loop ran (and consequently, data were captured) until the voltage reached the value set by the control 'final voltage display'. The 'true' case box with the $\geq$ symbol performed this comparison. At the end of the scan, the current and voltage arrays, 'current array' and 'voltage array', respectively, were assembled and passed to the main programme, STRIPDP.VI, for further manipulation. The STRIPGLOB.VI is a status monitor containing LEDs and timers to indicate the current step in the experimental procedure and the remaining time in this step. The data were saved in a spreadsheet file.

These data could be recalled using the LOADDP.VI processing VI. This VI smoothed the data and calculated the peak height and position after a proper selection of the baseline by cursor placement. A typical differential pulse voltammogram for rutin on the TCP-CPE electrode, after manipulation by the LOADDP.VI, is shown in figure 6.

The performance of the proposed system was tested by studying the electrochemical response of rutin. A study of the effect of pH, preconcentration time and preconcentration potential indicated that the optimum settings are pH 5.0, 120 s and 0.20 V accordingly. The precision of the method was checked by calculating the RSD of 10 replicate determinations on four samples containing 1.0 × $10^{-7}$–5.0 × $10^{-7}$ mol $l^{-1}$ of rutin. The RSD was in the range of 1.5–4.6%.

### Conclusions

The automatic systems described in this article, based on the Virtual Instrumentation concept, have a number of advantages over conventional systems. Experienced programmers may find it more difficult to initially convert to LabVIEW due to the different underlying logic and concepts associated with it. Newcomers to interfacing without any prior knowledge of programming techniques get used to VI concepts much faster because of the user-friendliness of the overall programming environment. However, there is no doubt that, once users familiarize themselves with the functionality of the various tools provided by the supplier, they will soon realize that developing control programmes is easier than working with conventional text-based languages. The LabVIEW software has excellent presentation and data manipulation capabilities, and programmes are modular and easily adaptable to different types of analysis. When the instrumentation developed in this work was applied to electrochemical experiments, it displayed excellent stability and consistency both for instrument control and data capture and analysis, leading to an enhancement of precision and accuracy.

### Acknowledgment

This work was supported financially by a research grant from the Greek Ministry of Energy and Technology.

### References

1. National Instruments Corporation, 1996, *LabVIEW User Manual.*
2. National Instruments Corporation, 1996, *LabVIEW Data Acquisition Basics Manual.*
3. Gostowski, R., 1996, *Journal of Chemical Education*, **73**, 1103.
4. Drew, S., 1996, *Journal of Chemical Education*, **73**, 1107.
5. Muyskens, M., Glass, S., Wietsma, T. and Gray, T., 1996, *Journal of Chemical Education*, **73**, 1112.
6. Bard, A. J. and Faulkner, L. R., 1980, *Electrochemical Methods* (NY: John Wiley), p. 565.

